# Arginine rich short linear motif of HIV-1 regulatory proteins inhibits Dicer dependent RNA interference

**DOI:** 10.1186/1742-4690-10-97

**Published:** 2013-09-11

**Authors:** Sanket Singh Ponia, Sakshi Arora, Binod Kumar, Akhil C Banerjea

**Affiliations:** 1 Virology Lab II, National Institute of Immunology, Aruna Asaf Ali Marg, New Delhi, 110067, India

## Abstract

**Background:**

Arginine Rich Motif (ARM) of HIV-1 Tat and Rev are extensively studied linear motifs (LMs). They are already established as an inefficient bipartite nuclear localisation signal (NLS). The unusual passive diffusion of HIV-1 NLS tagged reporter proteins across the nucleus is due to an unknown competing functionality of ARM. Recent findings about the role of retroviral proteins as a suppressor of RNA interference (RNAi) involving their basic residues hint an interesting answer to this alternate functionality. The present work explores the role of HIV-1 ARM as a uniquely evolved viral motif to combat Dicer dependent RNAi.

**Results:**

We show that RNA binding ARM of both HIV-1 Tat and Rev is a LM with a pattern RXXRRXRRR unique to viruses. Extending the *in silico* results to wet lab, we proved both HIV-1 Tat and Rev can suppress Dicer dependent RNA silencing process involving ARM. We show, HIV-1 Tat and Rev and their corresponding ARM can bind the RISC loading complex (RLC) components TRBP and PACT confirming ARM as an independent RNAi suppression motif. Enhancement of RNAi in infection scenario through enoxacin increases HIV-1 replication as indicated by p24 levels. Except Dicer, all other cytoplasmic RNAi components enhance HIV-1 replication, indicating crucial role of Dicer independent (Ago2 dependent) RNAi pathway in HIV-1 infection. Sequence and structural analysis of endo/exo-microRNA precursors known to be regulated in HIV-1 infection highlights differential features of microRNA biogenesis. One such set of miRNA is viral TAR encoded HIV-1-miR-TAR-5p (Tar1) and HIV-1-miR-TAR-3p (Tar2) that are known to be present throughout the HIV-1 life cycle. Our qPCR results showed that enoxacin increases Tar2 miRNA level which is interesting as Tar2 precursor shows Ago2 dependent processing features.

**Conclusions:**

We establish HIV-1 ARM as a novel viral motif evolved to target the Dicer dependent RNAi pathway. The conservation of such motif in other viral proteins possibly explains the potent suppression of Dicer dependent RNAi. Our model argues that HIV-1 suppress the processing of siRNAs through inhibition of Dicer while at the same time manipulates the RNAi machinery to process miRNA involved in HIV-1 replication from Dicer independent pathways.

## Background

Short Linear Motifs (also known as SLiMs, Linear Motifs or mini motifs) are short stretches of protein sequence which mediate protein-protein interaction [1]. At the molecular level, linear motifs (LMs) are modular units that are separate from other functional properties of protein molecule. They are typically short linear peptides of around 3-12 amino acids with a particular sequence pattern where few amino acid are fixed while others allow variations [2]. In general, LM imparts specific recognition and targeting activities (via domain-motif interactions) to molecule in which they occur [3]. In a cellular context, LMs are involved in protein-protein interactions, protein trafficking, and posttranslational modifications [4]. The short generation time and high mutation rate of RNA genome in association with natural selection has led to evolution of SLiMs that rewire cellular machinery [5,6]. Poor conservation over long evolutionary distances and plasticity of LMs make them a widely used functional module in pathogenic viruses such as Human Immunodeficiency Virus type 1 (HIV-1) [5].

Peptide regions from HIV-1 Tat and Rev have been identified as independent protein modules. For example, arginine rich motif (ARM) (residue 48-60 in Tat and residue 34–50 in Rev) is one such prototype of protein modules that are functionally active as cell-penetrating peptides (CPP) [7]. It was observed that inverse and retro forms of HIV-1 ARM are fully functional and ARM mediated delivery of cargo inside the cell is dependent on its size [8]. ARM of Tat and Rev in its physiological context binds cis-acting RNA elements like Transactivation Responsive RNA (TAR) and Rev Responsive Element (RRE) of viral genome respectively. Conventionally, the viral ARM mediates specific RNA recognition and is also considered as non-classical bipartite variant of the positively charged nuclear localization signal (NLS). HIV-1 ARM binds both importin α and importin β *in vitro*[9], although ARM mediated nuclear export is silenced *in vivo*[10]. The inefficient transport property of HIV-1 NLS is due to the existence of two competing functionalities within the same basic sequence stretch of viral ARM [11,12]. Interestingly, these importin binding properties of viral ARM (RKKRRQRRR) can be differentially altered by substituting the last three arginine residues of the ARM to glycine residues (RKKRRQGGG). Such mutation restores the importin α binding preferentially over importin β as is the case of effective NLS (SV40 NLS) [9,11,12]. Some reports also suggest the overwhelming binding affinity of the ARM towards negatively charged biomolecules (e.g. RNAs) as a reason for effective inhibition of the import complex *in vivo*[10] while others suspected cytoplasmic proteins as a competitor of the import carriers [13]. Presence of three additional terminal arginine residues in an otherwise effective NLS, raises many questions including what is the alternate cytoplasmic functionality of arginine rich variant of NLS, why a strong NLS has been attenuated into bi-functional LM in HIV-1 and how a weak NLS provides replication benefit to virus?

RNA Interference (RNAi) or gene silencing is an important cellular pathway that regulates eukaryotic gene expression and is also involved in innate immune response against viral infections [14]. Recently, several viral mechanisms for escaping antiviral RNAi has been discovered which includes suppression of RNAi, mutational escape from RNAi and modulation of the cell’s microRNA (miRNA) profile [15]. Similar to plant and insect viruses, several mammalian viruses encoding RNAi silencing suppression (RSS) function were reported [16]. These RNAi suppressors are multifunctional proteins that were previously shown to block innate antiviral immune responses involving the interferon (IFN) pathway [14]. Characteristics like early phase expression, non-structural nature and the cytoplasmic localization property of viral proteins has been cited for potent RNAi suppression [17]. Many concerns have been raised regarding the specificity and physiological relevance of putative RNAi suppressor as all of these proteins also have other well-defined and essential functions [18]. It has been suggested that the first step to determine the precise role of RSS activity in the viral life cycle is to identify the specific functional module associated with RNAi suppression in viral polypeptides [17]. Although analysis of SLiM from animal viruses which inhibits RNAi is yet to be reported, but it has been shown that heterologous suppressor proteins that inactivates RNAi-mediated host defences shows dsRNA binding functionality [19]. A classic example of RSS associated LM has been reported in plant viral proteins containing GW/WG motifs that play a critical role in disabling RNA-induced silencing complex (RISC) by sequestering Ago protein [20,21].

HIV-1 uses two different strategies to avoid RNAi mediated inhibition which are induced and intrinsic [22,23]. Induced RNAi resistance involves the selection of mutations in the target sequence or mutations outside the target sequence (point mutations, deletion) that induce a new RNA structure blocking the RNAi inhibition. Intrinsic RNAi resistance utilizes Tat to abrogate the ability of Dicer that results in inhibition of siRNA maturation [24-26]. Interestingly HIV-1 Rev homologue, HTLV-1 Rex has also been shown to interact with Dicer, suppressing RNAi silencing [27]. Strikingly, in both the studies, the retroviral proteins’ (Tat and Rex) arginine rich region was involved in interaction with Dicer. Hence a detailed analysis of RNA binding viral ARM offers a testable hypothesis which can correlate Dicer dependent RNAi suppression with the unknown cytoplasmic functionality of HIV-1 ARM. However, it is still important to know the physiological relevance of RSS activity for a virus such as HIV-1 which is known to modulate microRNAs pathway for a successful replication [28]. So far there is a poor evidence of global RNAi suppression in HIV-1 infection, which conflicts with predicted RSS activity of its suppressor ARM. In fact, endogenous microRNAs pathway is still operative that could process both viral and cellular precursor miRNA to enhance HIV-1 replication [29,30]. So an explaination is needed for the biogenesis of microRNAs in case of HIV-1 infection that blocks the key central enzyme Dicer through Tat [24] and also downregulates Dicer expression in macrophages through Vpr [31]. Inhibition of Dicer both at RNA and protein level is a general trend by many mammalian viruses [32,33]. Recently, Ago2 dependent dicing activity has been discovered establishing Dicer independent RNAi biogenesis [34,35]. This non-canonical pathway plays a role in the production of non-coding RNA such as piRNA for transposon regulation [36] and miRNA for regulating gene expression [37]. However, its role in host-pathogen interaction and modulation of cytoplasmic RNAi to selectively favor the demands of HIV-1 is unknown.

In the present work, we have established the identity of HIV-1 ARM as a unique viral motif which targets the RNAi silencing pathway. Using bioinformatics we define the RNA binding region (ARM) of HIV-1 Tat and Rev as a SLiM unique to viruses. Interactome network maps of HIV-1 Tat and Rev were analysed to deduce the common interacting partners that can be uniquely attributed to the viral ARM. Using the computational approach we predicted that ARM could possibly target RNAi pathway. We produced experimental evidences for the RSS activity of ARMs in HIV-1 proteins. Using transient expression assays that use Dicer dependent RNA silencing of reporter gene followed by reversal of silencing by suppressor proteins, we identified the RSS activity of HIV-1 Rev that works in additive fashion with HIV-1 Tat. We also demonstrate the role of ARM in the suppression activity of these HIV-1 proteins in the reversal-of-RNAi-silencing assay. Furthermore, we confirm the independent nature of ARM as a LM sufficient to confer gain of RSS functionality to reporter proteins. We go on finding the mechanistic detail of this RNAi suppression motif by investigating its critical role in targeting of RLC components by HIV-1 regulatory proteins. We also prove that HIV-1 ARM can bind RNAi components autonomously confirming its modular nature. To resolve the discrepancies reported in current literature, we have analyzed the effect of RNAi and its components on HIV-1 replication using indicative p24 levels. Our results suggest that RNAi in its physiological niche of HIV-1 infection and all its key cytoplasmic components except Dicer, positively modulate HIV-1 replication.

## Results

### ARM of Tat and Rev carries a SLiM with a pattern RXXRRXRRR unique to viruses

**Figure 1:**
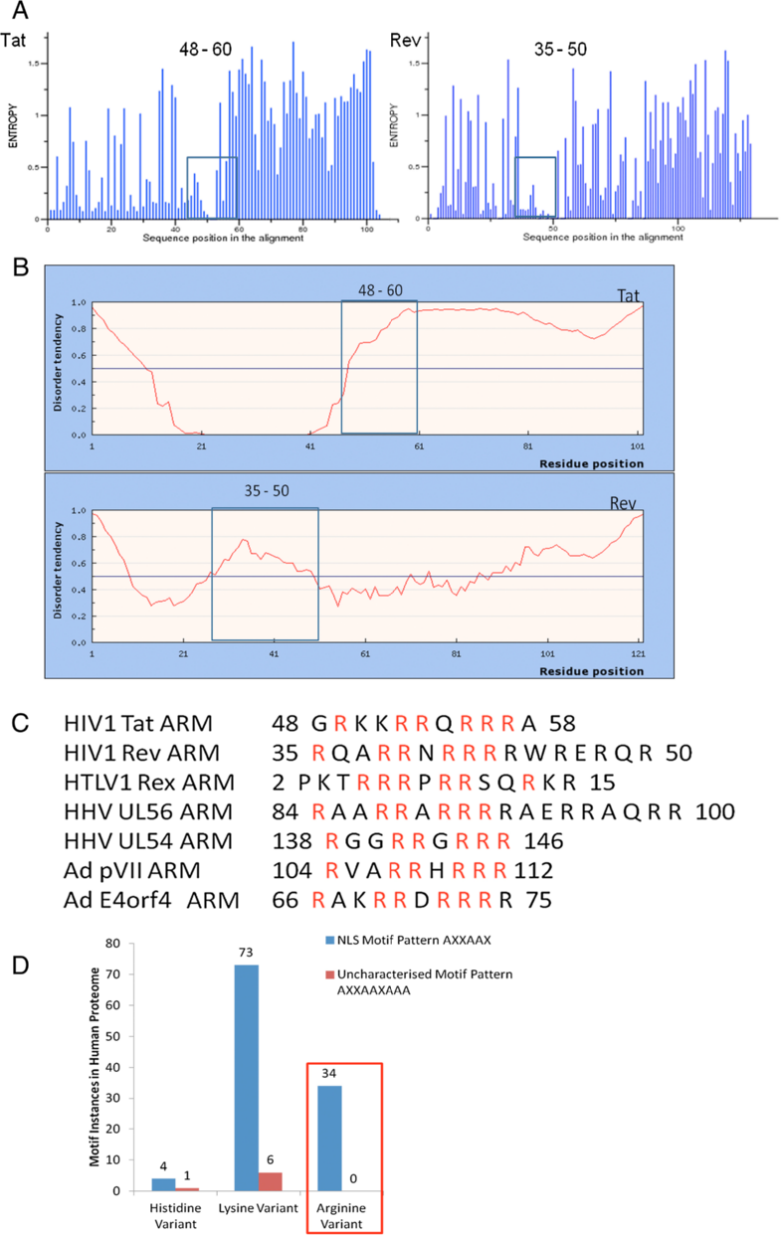
**Linear Motif discovery based on motif definition.** To discover linear motif (LM), multiple sequence alignment (MSA), Shannon entropy plots and sequence context in terms of structural disorder were used. **(A)** shows the Shannon entropy plot of HIV-1 Tat and Rev to analyze variation in sequence conservation. This plot quantifies diversity using MSA at each single amino acid position in full length protein. Amino acid positions that do not exhibit any changes in protein sequences have entropy of zero whereas a position that shows highly variable substitution is represented by large peaks. The basic region of both Tat and Rev with a motif pattern RXXRRXRRR is present in regions that show more than 90% conservation indicating their importance in viral genome. Disorder predictions using IUPred plot **(B)** indicates that Arginine Rich Motif (ARM) in both Tat and Rev is above the threshold of 0.5 predicting their nature as natively disordered polypeptide. **(C)** shows the sequence comparison of the ARM in different viral proteins of Human immunodeficiency virus type 1 (HIV-1), Human T-lymphotropic virus (HTLV1), Human herpes virus (HHV) and adenovirus (Ad). Tat, Rev, Rex, UL56, UL54, pVII, E4orf4 are viral open reading frames and their corresponding ARM are shown with arginine residues highlighted in red. The numbers identify residue positions in full length proteins. **(D)** shows the distribution of basic amino acids in two LM patterns in the host proteome. One is AXXAAX that is a well defined classical NLS pattern of host while AXXAAXAAA is an uncharacterized viral LM. This analysis highlights the specificity of ARM from viral proteins as a Short linear motif (SLiM).

Linear motifs (LMs) are 3-12 amino acid long peptides of conserved sequence and flexible structure. This definition was applied to a consensus of 170 protein sequences each of HIV-1 Tat and Rev from subtype references of 2010 HIV sequence compendia. Shannon entropy plots (Figure 1A) of both HIV-1 Tat and Rev multiple sequence alignment (MSA) reveal relative conservation of motif pattern RXXRRXRRR at N-terminus region. The ARM of HIV-1 Tat is in residue position 48–60 while that of HIV-1 Rev is in position 35–50. To validate arginine rich sequence as a LM, we analyzed flexibility of motif pattern and its embedding structural context using disorder profile. The IUpred score that shows the high flexibility index with a cut off of 0.5 shows the motif to be present in disordered region of both the Tat and Rev proteins (Figure 1B). The conservation of ARM with pattern RXXRRXRRR in other viral proteins (Figure 1C) further indicate that ARM can act as a viral motif. To check the molecular mimicry of viral ARM with host motifs we compared the distribution of basic amino acids in two linear motif patterns in the host proteome (Figure 1D). One is AXXAAX that is a well defined classical nuclear localization signal (NLS) pattern of host while AXXAAXAAA is an uncharacterized viral LM. A can be any of the three basic amino acids and X any amino acid. RXXRRXRRR shows zero instances in human proteome confirming the evolution of ARM exclusive to the viruses.

### ARM could target proteins involved in nucleic acid based immunity

**Figure 2:**
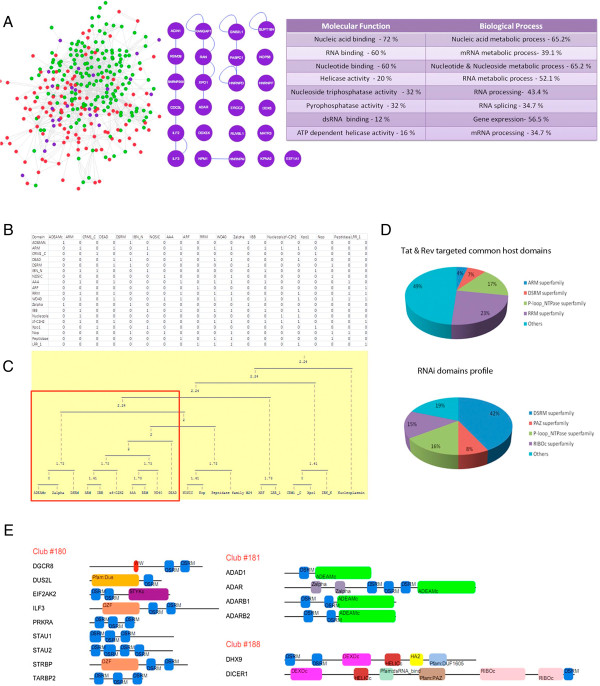
**Bioinformatics based functional assessment of HIV-1 ARM.** To assess the functional role of viral ARM, molecular interaction networks for HIV-1 Tat and Rev proteins was constructed using HIV-1 interaction database **(A)**. The Tat targeted host proteins are in green colour, Rev targeted in red and common interacting partners in purple. Individual proteins are depicted as nodes and edges represent interactions. The functional assignment of the commonly targeted proteins are according to gene annotation (GO) (molecular function and biological process) and is summarised with top 8 GO term with p value <10^-7^ along with their cluster frequency as percentage. **(B)** shows domain profiling of commonly targeted host proteins by ARM is based on correlated domain structures. In the matrix representation, both rows and columns are domains of targeted proteins, “1” and “0” represent the presence or absence of homo or heterotypic domain interaction among them respectively. Single linkage hierarchial clustering **(C)** based on Euclidean distance gives clusters of domains (domain clubs) as a possible protein context that can be targeted by ARM containing proteins. The red box indicates domain clubs shared by pathways of nucleic acid based on immunity. **(D)** shows the most represented structural domains in commonly targeted host proteins and RNAi pathway. In both the pie chart, Double-stranded RNA binding motif (DSRM) and P-loop_NTPase (AAA domain) are common and enriched. Based on these domains and the club to which they belong, most probable targets of viral ARM are shown in **(E)**. Refer method for details.

To deduce the physiological function of ARM, molecular interaction network for Tat and Rev was constructed using HIV-1 interaction database. The interacting partners for both HIV-1 Tat and Rev are shown in Figure 2A. As proteins with identical motif show similar interactome profiles, we assessed functional classification of 25 common interacting partners of Tat and Rev. The best represented molecular and biological function involved RNA binding and mRNA metabolic process that can be attributed to the presence of ARM. Most SLiMs provides gain of interaction function through domain-motif interaction. To find the ARM targeted protein domains, we carried out domain profile analysis of commonly targeted proteins from Figure 2A. The common interacting proteins are decomposed into their functional units (domains) and clustered according to their known domain-domain interactions (Figure 2B and Figure 2C). The domain profiles (Figure 2D) of common interacting partners and RNA Interference (RNAi) proteins (Drosha, DGCR8, XPO5, RAN, DICER1, DHX9, TRBP, PACT, Ago2) highlighted that both dataset are enriched for DSRM and P-loop_NTPase (AAA) domain superfamily. Using these protein domains as functional units, we explored possible domain clubs (proteins) that are probable interacting partners of ARM. Figure 2E shows the predicted host proteins that could interact with ARM are involved in nucleic acid based immunity including RNAi.

### Viral proteins can suppress Dicer dependent RNA silencing process using ARM

**Figure 3:**
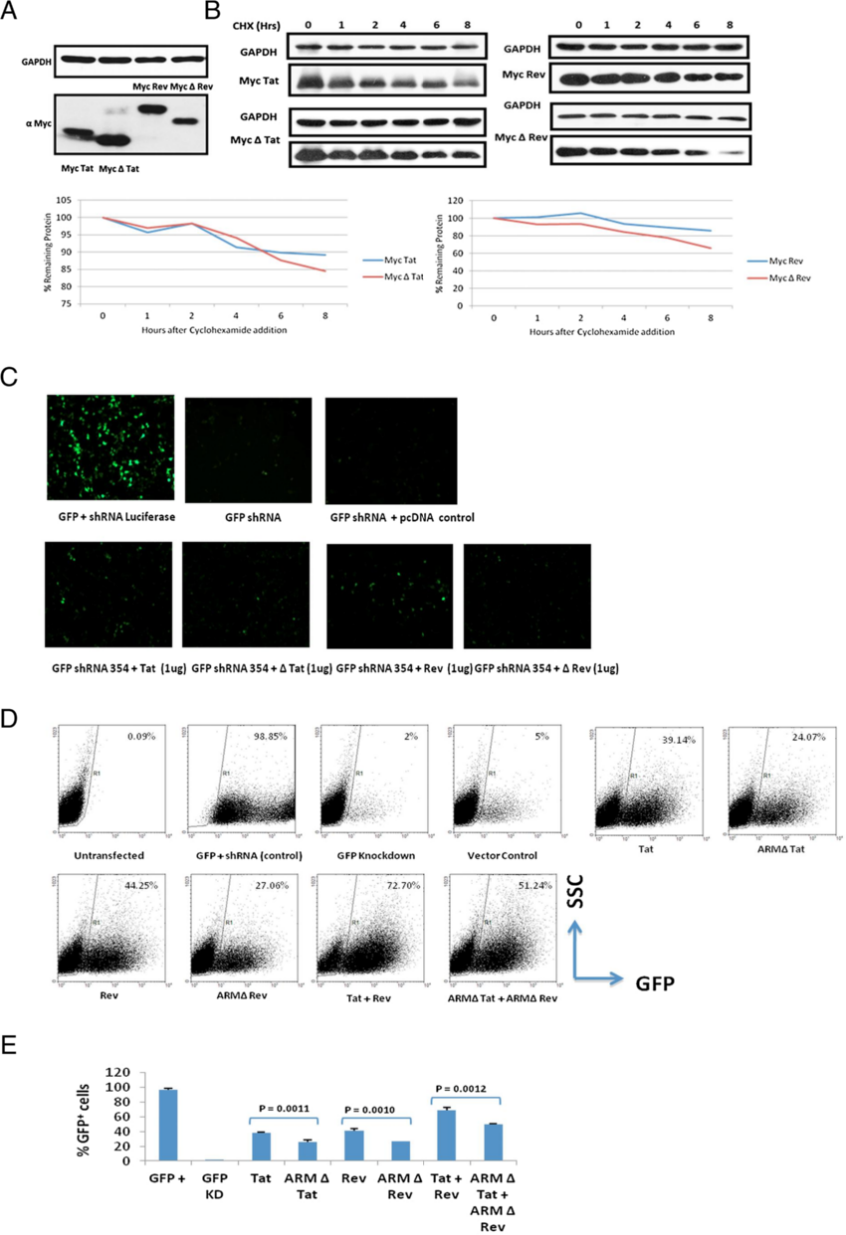
**HIV-1 Tat and Rev can suppress Dicer dependent RNAi process involving ARM.** HEK 293 T cells were transfected with an expression plasmid for HIV-1 Tat, Rev and their corresponding ARM deletion mutants i.e. ∆Tat and ∆ Rev (1 μg each). In ARM mutants the ARM region was deleted (48-60 in Tat and 35-50 in Rev). **(A)** shows the expression levels of wild type and ARM mutants of Tat and Rev respectively. The stability of Tat, Rev and their corresponding ARM deletion mutants was determined using Cycloheximide chase for 8 hours **(B)**. HEK 293 T cells were co-transfected with expression plasmid for GFP, Dicer dependent shRNA against GFP (0.5 μg) along with Tat, Rev and their corresponding ARM deletion mutants. GFP expression was measured at 48 hours post transfection. **(C)** shows fluorescence microscopic images of RNAi suppression by HIV-1 Tat, Rev and their ARM deletion mutants. **(D)** is a Flow cytometric analysis to show the suppressor effect of HIV-1 Tat, Rev and ARM deletion mutants on GFP expression in Dicer dependent RNAi suppressor assay. Dot plot depicts the number of cells (counts) on Y-axis versus the expression of GFP reporter (FL1) on X-axis. **(E)** is a histogram plot of suppressor assay. The p values indicate that the difference between the wild type Tat/Rev and corresponding ARM mutant is significant.

ARM deletion mutants of HIV-1 Tat and Rev were cloned with Myc tag and their expression was checked (Figure 3A). Figure 3B shows that there is no significant change in the stability of Myc tagged Tat and Rev and their respective deletion mutants. To investigate the role of ARM in the RSS function, Tat, Rev and their respective deletion mutants were used in the reversal-of-Dicer-dependent-silencing assay based on GFP reporter. Figure 3C shows that both Tat and Rev can act as RSS by reverting the knockdown of GFP as seen in fluorescence microscopy images (Figure 3D) while deletion of ARM compromised RSS activity. The same results were confirmed by flow cytometry (Figure 3E). The RSS effect of HIV-1 Tat and Rev was additive in nature. In addition NS4B, a Dengue viral protein that has a similar ARM as that of HIV-1, shows RSS activity. Deletion of ARM in NS4B compromises the RSS function (Additional file 1: Figure S1).

### HIV-1 ARM independently confers Dicer dependent RNAi suppressor function

**Figure 4:**
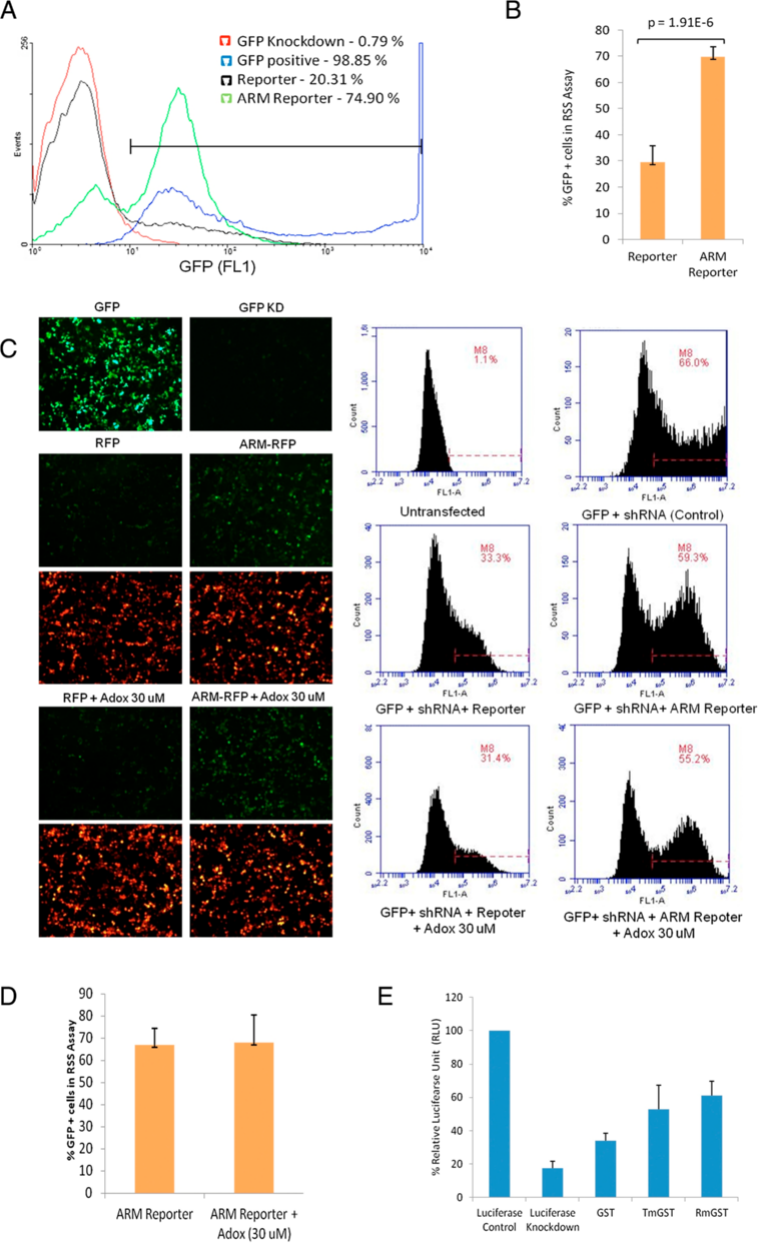
**HIV-1 ARM independently confers Dicer dependent RNAi suppressor function.** ARM was fused to a reporter protein RFP (ARM RFP). HEK 293 T cells were co-transfected with an expression plasmid for GFP and Dicer dependent shRNA against GFP (0.5 μg) along with RFP vector/ARM RFP. **(A)** shows a histogram plot (Events on Y axis and expression of GFP (FL1) on X axis) of suppressor assay (as described in 3D) where RSS activity is independently conferred by fusion of ARM to the reporter protein (RFP). The bar graph in **(B)** shows the quantification of the histogram. **(C)** shows flow cytometric analysis (Histogram plot showing number of cells (counts) on Y-axis versus the expression of GFP reporter (FL1) on X-axis) and corresponding fluorescence microscopy images of the suppressor assay of ARM-Reporter in the presence and absence of 30 μM Adox (Adenosine, periodate). The bar graph in **(D)** shows the quantification of the histogram. **(E)** show the ARM mediated reversal of Dicer-dependent-silencing assay based on luciferase reporter with a significant p value (< 0.001).

In order to show that ARM independently confers RNAi suppressor function it was fused to a reporter protein RFP (ARM RFP). Figure 4A shows that ARM is sufficient to provide more than 2 fold suppressor activity to ARM RFP as compared to control hence proving that HIV-1 ARM can independently confer RNAi suppressor function. Figure 4B shows quantification of 4A. To investigate the role of methylation in ARM mediated RNAi suppression, Adox (a general methylation inhibitor) was used in the reversal-of-Dicer-dependent-silencing assay based on GFP reporter. Results (Figure 4C) show that ARM mediated RNAi suppressor function is independent of arginine methylation. Figure 4D shows the quantification of flow cytometry in 4C. The same results were confirmed using Dicer-dependent-silencing assay based on luciferase reporter (Figure 4E).

### HIV-1 Tat, Rev and ARM can bind TRBP and PACT

**Figure 5:**
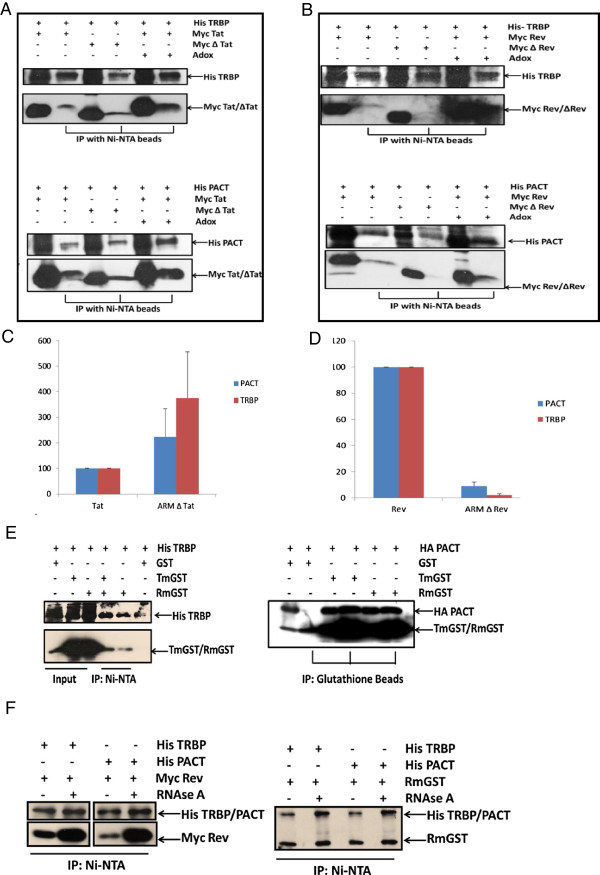
**HIV-1 ARM confers interaction ability with components of RISC Loading Complex (RLC).** HEK 293 T cells were co-transfected with His TRBP, His PACT and Myc Tat, Myc Rev, and their corresponding ARM deletion mutants in the presence and absence of Adox. 48 hours later cell lysates were subjected to co-immunoprecipitation as described in methods. TRBP and PACT immunoprecipitates were assessed by western blotting using anti-His and co-immunoprecipitated Tat, Rev and their ARM deletion mutants were detected using anti-myc **(A** and **B)**. **(C)** and **(D)** show the relative binding of ARM deleted mutants with respect to wild type proteins with TRBP and PACT respectively. **(E)** shows co-immunoprecipitation of His-TRBP and HA-PACT with GST and GST fused HIV-1 ARM (TmGST and RmGST) using a similar protocol as in 5A (using Ni–NTA in first case and Glutathione Beads in second). **(F)** is co-immunoprecipitation analysis of His TRBP and His PACT with Myc Rev and RmGST in the presence and absence of 50 μg/ml of RNase A.

As suggested by our *in silico* results (Figure 2), the probable mechanism of ARM to act as Dicer dependent RNAi suppressor is possibly through interaction with proteins which are enriched in Double-stranded RNA binding motif (DSRM) and/or P-loop_NTPase domains. To examine this possibility, we evaluated the ability of HIV-1 Tat and Rev, their respective ARM deletion mutants and ARM containing reporter protein to bind components of RISC loading complex (TRBP and PACT as both constitutes DSRM in their structure). As shown in co-immunoprecipitation results (Figure 5A and Figure 5B), both wild type Tat and Rev are able to bind TRBP and PACT whereas their respective ARM deletion mutants shows differential binding ability. Tat ARM mutant was fully functional in binding both TRBP and PACT while Rev ARM mutant showed compromised TRBP and PACT binding. The binding of Tat and Rev also remains intact even in the presence of methylation inhibitor (Adox) suggesting RNAi suppression is independent of the methylation status of suppressor proteins (Figure 5A and Figure 5B). Figure 5C and 5D shows the relative quantification plot for 5A and 5B respectively. To confirm ARM as an RNAi suppression motif we also showed its ability to independently confer gain of TRBP and PACT binding functionality to reporter GST protein. As shown in Figure 5E, both Tat and Rev ARM fused GST (TmGST and RmGST respectively) can bind TRBP and PACT whereas GST alone fails to do so. To address whether such protein interactions require RNA as a mediator, the cell extracts were treated with RNase-A prior to co-immunoprecipitation. Figure 5F shows that wild type Rev is able to bind TRBP and PACT even in the absence of RNA. RmGST also shows similar results with TRBP and PACT. RNAse-A treatment substantially increases this binding probably because of the competitive RNA binding by ARM containing proteins. As this competition is diminished by RNAse-A treatment, more ARM could be rapidly available for interaction with host proteins.

### RNAi affects HIV-1 replication

**Figure 6:**
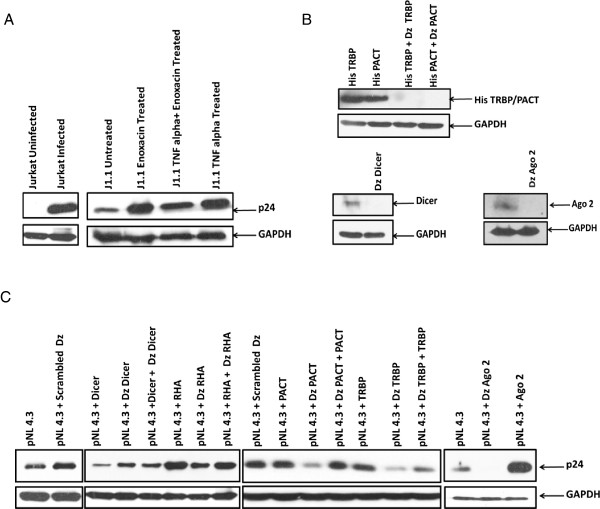
**RNAi affects HIV-1 replication.** Latently infected T cells (J1.1) were treated with TNF-α (10 ng/ml) and enoxacin (50 μM) for 48 hours. **(A)** shows p24 levels by western blot on enoxacin and TNF-α treatment. **(B)** shows use of 10-23 DNAzyme in sequence specific knockdown of RLC components PACT, TRBP, Dicer and Ago2. **(C)** shows the levels of p24 on Dicer, RHA, PACT, TRBP and Ago2 knockdown (DNAzyme)/Overexpression and both. GAPDH was used as a loading control.

To establish the role of cellular RNAi in HIV-1 infection, latently infected T-cells (J1.1) were treated with enoxacin (TRBP dependent RNAi enhancing drug) [38,39]. The drug treatment at 50 μM for 48 hours reactivates latent HIV-1 which is comparable to TNF-α (10 ng/ml, a known activator of HIV-1 latency) as shown by p24 western blots (Figure 6A). This suggests a positive co-relation of RNAi and HIV-1. To check the effect of cytoplasmic components of RNAi on HIV-1 replication, p24 levels were measured in over expression and DNAzyme mediated knockdowns of cytoplasmic RNAi components. Co-transfection of pNL4.3 (HIV-1 molecular infectious clone) along with PACT, TRBP, RHA, Dicer and Ago2 with respective DNAzyme (for sequence specific knockdowns, Figure 6B) was carried out and p24 levels were analyzed using western blotting. Figure 6C shows that except Dicer, other cytoplasmic RNAi components promote HIV-1 replication. The effect was specifically reversed by DNAzyme mediated knockdown.

### MicroRNA profile in HIV-1 infection suggests differential role of Dicer dependent and independent RNAi

**Figure 7:**
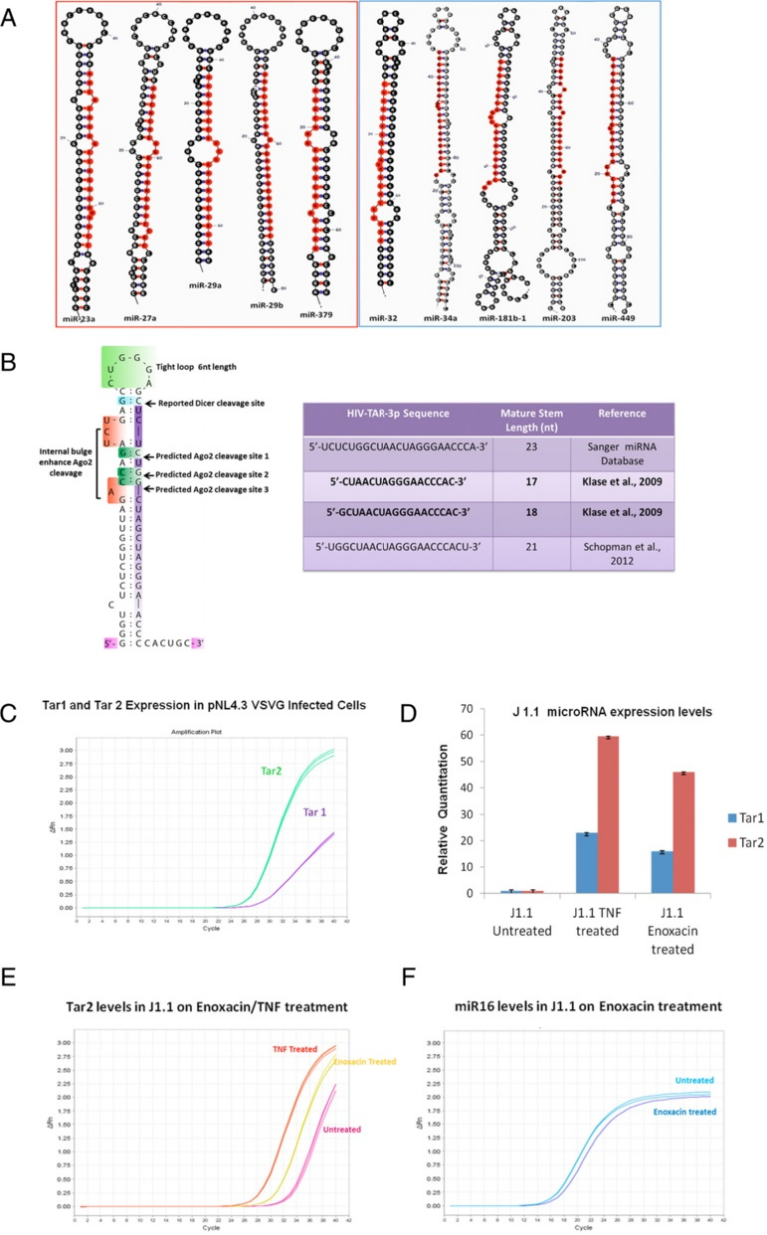
**MicroRNA profile in HIV-1 infection suggests differential role of Dicer dependent and independent RNAi. (A)** shows the distinct structure pattern in the terminal loop of hairpin precursor of microRNA known to be up-regulated (Blue Box) and down-regulated (Red Box) in HIV-1 infection. Up-regulated miRNAs show Ago2 dependency while down-regulated show Dicer dependency. Sequences in red represents mature miRNA in hairpin precursor. **(B)** the left half shows Ago2 processed molecular features in TAR miRNA (short tight loop in green box), internal bulges (in red), and predicted cleavage sites by RNA endonucleases. These predictions were based on the functional parameters of Ago2 dependent processing reported in literature [35,36,39,40]. The right half shows the TAR2 miRNA sequence with mature stem length of 17 to 18 (in bold) as predicted to be dominantly processed by Ago2. Total RNA was prepared form the HIV-1 infected cells and subjected to Real Time PCR to analyze the expression level of Tar1 and Tar2 miRNA. **(C)** shows the amplification plot for Tar1 and Tar2. Latently infected T cells (J1.1) were treated with TNF-α (10 ng/ml) and enoxacin (50 μM) for 48 hours. Total RNA was prepared and was subjected to Real Time PCR to analyze the expression level of Tar1 and Tar2 miRNA. **(D)** shows the quantification plot for Tar1 and Tar2. **(E)** shows the amplification for Tar2. **(F)** shows the amplification plot for miR16 (an unrelated miRNA) in enoxacin treated and untreated samples. RNU6B was used as endogenous control.

To analyze the Dicer dependent and Dicer independent RNAi in HIV-1 infection, we assessed Dicer and Ago2 processed microRNA signature pattern in precursor microRNA (using miRNAMap, a structural database of miRNAs) that are known to be differentially regulated in HIV-1 infection. Sequence/structure pattern in microRNA terminal loop shows distinct molecular features suggesting different pathway of microRNA biogenesis in HIV-1 replication. Figure 7A shows Dicer dependent features in hsa-miR-23a, 27a, 29a, 29b and 379 (that are known to be inhibited in HIV-1) while Ago2 dependent molecular features of miRNA biogenesis was evident in hsa-miR-32, 34a, 181b-1, 203 and 449 (that are known to be up regulated in HIV-1 infection). To check whether exogenous viral microRNA can adapt to differential microRNA biogenesis in HIV-1 infected cells, sequence and structural features of TAR precursor microRNA was analyzed. Figure 7B shows that TAR microRNA precursor carries Ago2 processed features that predict the maturation of only HIV-TAR-3p (TAR2). A quantitative PCR of TAR1 (miR-TAR-5p from 5’ arm) and TAR2 (miR-TAR-3p from 3’ arm) processed mature miRNA was also done in infection based scenario (Jurkat cells) and in enoxacin treated latently infected J1.1 T cells. qPCR in VSV-G pseudotyped HIV-1 infected T-cells shows expression of TAR2 is predominant over TAR1 (Figure 7C). Similar result were found when latently infected T cells were activated by enoxacin treatment while a negative control (hsa-miR16) shows no change (Figure 7, panel D-F).

## Discussion

RNA interference (RNAi) is a natural process whereby double-stranded RNA (dsRNA) induces sequence-specific degradation of RNA. It is the predominant mechanism of antiviral defence in plants and invertebrates but in mammals RNAi chiefly regulates host cellular processes through miRNA pathway and restricts transposable elements [15]. In vertebrate cells, there has been evolutionary progression from RNA-based immunity to protein-based immunity [40,41]. This is also evident from the phylogenetic analysis and evolutionary conservation of RNAi components. For example, plants reveal multiple homologues of dsRNA-binding Dicer while mammals show only one [42]. The reason of such drastic shift from RNA to protein based innate immunity in vertebrates is not clear. We hypothesized that HIV-1 (along with other Retroviruses) might enocde an unidentified Dicer dependent RNAi suppressor function that counteracts antiviral RNAi and host strikes back by negatively selecting antiviral RNAi.

Plant viruses are known to exploit short linear motifs (SLiMs) by acquiring GW/WG peptides in viral ORFs which binds to Ago proteins making them incompetent in targeting viral RNA. However, no such generalized linear motif (LM) is reported in case of any mammalian virus that can be associated with the modulation of RNAi surveillance. In the present work, we started with bioinformatics analysis that predicts HIV-1 Arginine Rich Motif (ARM) as a unique viral motif that targets the nucleic acid based immunity including RNAi pathway. Using Dicer dependent RNAi silencing suppressor (RSS) assay the suppression activity of HIV-1 Tat and Rev, their corresponding ARM deletion mutants and ARM fused reporter proteins were measured. HIV-1 Tat and Rev both can suppress Dicer dependent RNAi silencing process while the ARM deleted mutants show compromised RSS activity clearly indicating the importance of ARM. Furthermore, we have shown that the HIV-1 ARM alone was sufficient to suppress RNAi and this functionality was independent of its methylation status. We investigated the critical role of ARM in targeting components of RISC Loading Complex (RLC). Both HIV-1 Tat,Rev were able to bind TRBP/PACT and the binding was not abolished in the presence of general methylation inhibitor (Adox) [43]). Adox appears to increase this binding which could be due to the absence of methylation on arginine residues that are now easily available to interact more strongly with TRBP/PACT. However, deletion of ARM alters the RLC binding. ARM deletion mutant of Tat showed increased relative binding which can be explained on the basis of its structure which is unusually sticky in nature. While ARM delta Rev failed to bind TRBP/PACT as expected. We further assessed the functionality of viral ARM using novel chimeric proteins. In the co-immunoprecipitation, we found that ARM confers a gain of interaction function to the reporter protein. Hence, we prove that HIV-1 ARM can bind RNAi components independently confirming its modular nature.

To understand the role of cytoplasmic RNAi in the HIV-1 replication, we used RNAi enhancing drug (enoxacin) in HIV-1 latently infected T cells. Enoxacin is shown to enhance RNAi processing of RISC specifically through TRBP [38]. Our results showed that enoxacin treatment reactivates latently HIV-1 infected T cells which is comparable to a known latency reactivator i.e., TNF-α. To gain an insight in the mechanism of enoxacin dependent increase in p24 levels of latently infected T-cells, we analyzed the effects of RNAi components on HIV-1 replication. Except Dicer, all other cytoplasmic RNAi components (RHA, PACT, TRBP and Ago2) were shown to positively modulate HIV-1 replication as indicated by p24 western blots. Our studies show the role of HIV-1 ARM as a general suppressor of Dicer dependent RNAi. But at the same time we observe a positive correlation between RNAi and HIV-1 replication as measured by p24 western blotting after enoxacin treatment. This apparent paradox is resolved by careful assessment of the microRNA biogenesis in the HIV-1 infection. The RLC comprises of 4 protein components: Dicer, PACT, TRBP and RHA. The RLC is involved in the activation of RNA induced silencing complex (RISC) which uses siRNA to degrade the viral genome that is known as Dicer dependent RNAi. RISC is also involved in the processing of miRNA from precursor miRNA. These miRNAs are known to be differentially expressed (at the transcription level) in HIV-1 infection. Recently, Dicer independent activation of RISC and miRNA processing has been shown [35]. To understand the role of Dicer dependent and independent pathways in HIV-1 replication, we analysed the differentially regulated miRNAs in HIV-1 infection [29,44-47] and their corresponding sequence/structure signatures that determines their biogenesis. Interestingly, the down-regulated microRNAs hsa-miR-23a, 27a, 29a, 29b and 379 and up-regulated microRNAs hsa-miR-32, 34a, 181b-1, 203 and 449 show different features in terminal loop. The terminal loop plays a very critical role in Dicer and Ago2 substrate specificity for microRNA biogenesis [48-50]. Down-regulated microRNAs show a large terminal loop that enhances pre-miRNA cleavage by Dicer. The up-regulated miRNAs shows a tight, short loop feature which is essential for Ago2 cleavage. As HIV-1 TAR2 also shows a similar loop feature, we predicted it to be processed by Ago2 in case of Dicer inhibition and show preferential release as already reported in literature [51]. Our qPCR results in infectious scenario confirmed these predictions. Similar results were seen when TRBP dependent RNAi was enhanced using enoxacin in latently infected T-cells. The presented data suggest a possible role of Dicer independent (Ago2 based) RNAi in HIV-1 infection. Ago2 is known to be associated with many more miRNAs including miR-30a, 21, 92a, 99b, 183, 27a, 151a, 30d, 182, 25 [52] that might influence HIV-1 replication. Also Ago2 is known to be essential for HIV-1 replication [53]. As interferon response and RNAi silencing in HIV-1 infection involves downregulation of Dicer [32] and possible upregulation of many Dicer independent microRNAs [54], further research on Ago2 processed endogenous and exo-miRNAs might explain the cross talks these two antiviral pathway play in modulating the HIV-1 replication. We hypothesize that Ago2 dependent miRNAs could also play a role in innate and adaptive immunity explaining why the evolutionary ‘fossils’ of RNAi components are still conserved in vertebrates for robust viral restriction.

## Conclusions

Our work explains that Dicer dependent RNAi restriction is suppressed in case of successful HIV-1 replication. We have established the case of HIV-1 ARM as an example of a viral module that can effectively manipulate the host restriction pathway. The presence of similar arginine rich linear motifs in other viral proteins (Additional file 1: Figure S1) suggests a general modus operandi for RNAi modulation. Now it is possible to explain the potent suppression of Dicer dependent RNAi pathway in other viruses based on the presence or absence of such ARM in their respective proteome.

## Methods

### Evolutionary conservation, disorder prediction & motif search

Sequence conservation analysis was based on multiple sequence alignment of HIV-1 Tat and Rev Protein sequences from Los Alamos HIV sequence database, HIV premade alignments (http://www.hiv.lanl.gov/content/sequence/HIV/mainpage.html). Shannon entropy plots were made using Shannon Entropy-One tool (http://www.hiv.lanl.gov/content/sequence/ENTROPY/entropy_one.html). The flexible structural context was analysed using disorder profile generated via IUPred (http://iupred.enzim.hu/). Protein BLAST search (blastP) was used for evaluating arginine rich motif (ARM) conservation in other viral protein (Viruses, taxid: 10239). SLiMSearch (http://bioware.ucd.ie/~compass/biowareweb/) was used to search the ARM in the context of human proteome. SLiMSearch was used to identify occurrences of these two defined motif patterns against human proteome using evolutionary conservation and structural disorder context statistics. Only those instances are counted that are above the cut off threshold (p = 0.001, IUP = 0.5). See these websites for details of bioinformatics analysis.

### Interactome generation and functional classification

The 246 unique interacting partners for HIV-1 Tat interactome has been taken from two sources. One is BioGRID (http://thebiogrid.org/) and the other from reference [55]. The 240 unique interacting partners for HIV-1 Rev are entirely taken from reference [56]. The molecular interaction network diagrams were generated by Cytoscape [57] using import network from table option. To make the interaction maps more informative, previously established host interactions were also integrated employing publicly available Protein-Protein Interaction database HPRD (http://www.hprd.org/). To examine molecular functions and biological processes of common host targets of HIV-1 Tat and Rev, a systematic gene annotation using BiNGO [58] was carried out on 25 host protein in cytoscape.

### Domain composition, domain profiling and protein target prediction based on domain clubs

To document the conserved structural domain profiles on 25 commonly targeted host proteins, we utilised batch search tool of conserved domain database (CDD). For every domain annotation, we counted the number of protein instances in the interactome dataset. All the top scorers in percentage were considered to be the most represented protein domains in the common interactome of HIV-1 Tat and Rev. The conserved structural domain profiles of common targets were than subjected to a domain-domain matrix to map the homo or heterotypic domain interaction among them using domine database (http://domine.utdallas.edu/cgi-bin/Domine). In the matrix representation “1” and “0” represent the presence or absence of interaction respectively. The domain interaction profiles were then clustered using single linkage hierarchical clustering based on Euclidean distances. The domain clusters were then used to predict the proteins as the possible target of ARM using domain club search. (http://pawsonlab.mshri.on.ca/DomainClub/domainClub.php).

### Plasmids, cell culture and transfection

RetroQ-ZsGreen vector (Clontech) co-expressing GFP under CMV promoter and Dicer processed shRNA against GFP under U6 promoter was used as reported in reference [14]. The HIV-1 Tat, Rev and their respective mutants (Delta ARM mutants, 48-60 in Tat and 35-50 in Rev) were cloned downstream of the CMV promoter in the pCDNA vector. They were also subcloned in Myc-Tag Vector from Clontech. Rev ARM motif was cloned as fusion with RFP using DsRed-Express Fluorescent Protein vector (Clontech). Tat and Rev ARM motif were also cloned as fusion with GST using pEBG vector. TRBP and PACT were cloned as a fusion with His tag using pCDNA vector and as HA-tagged using HA vector from Clontech. Dicer was cloned as a fusion with Myc tag using Myc vector from Clontech. pcDNA3-HA RHA construct was a kind gift from Dr. Toshihiro Nakajima (St. Mariana University school of Medicine, Japan). HA-Ago2 was procured from Addgene. The 10-23 DNAzyme sequence for knock down of TRBP, PACT, RHA, Ago2 and Dicer is provided in Additional file 2: Table S1. HEK 293 T (Human Embryonic Kidney 293 T) cells were maintained in DMEM (HiMedia) supplemented with 10% fetal calf serum, 100 U/mL penicillin and 100 μg/mL streptomycin (Invitrogen, California) at 37°C with 5% CO_2_. Jurkat E6.1 (Human T cell lymphoblast like cell line) and J1.1 (Latently infected T cells) cells were maintained in RPMI 1640 media (Himedia) supplemented with glutamine, 10% FCS, 100 U/mL penicillin and 100 ug/mL streptomycin (Invitrogen) at 37°C with 5% CO2. Plasmid transfections were performed using Lipofectamine 2000 (Invitrogen) or Jetprime (HiMedia) as per the manufacturer’s protocol.

### Dicer dependent RNAi suppression assay

A transient gene silencing system was used for assaying Dicer dependent RNAi response. This involves transient transfection of plasmids coding for GFP, its corresponding Dicer dependent shRNA and suppressor of RNAi in HEK 293 T cells (as described in [14]). 30 μM Adox (Adenosine, periodate) treatment was given to one set of cells. Suppression of GFP expression shows a healthy Dicer dependent RNAi process whereas its reverse shows a compromised RNAi response. Expression of GFP is analysed using fluorescence microscopy and quantified through flow cytometry.

### Immunoblotting

Relative levels of different proteins were compared by immunoblot analysis. Cells were lysed with RIPA lysis buffer (20 mM Tris [pH7.5], 150 mM NaCl, 1 mM Na_2_EDTA, 1 mM EGTA, 1% Triton X-100, 1% sodium deoxycholate, 2.5 mM sodium pyrophosphate, 1 mM beta-glycerophosphate, 1 mM Na_3_VO_4_). Protein estimation was done using BCA Protein Estimation Kit (Pierce Biotechnology Inc.). Proteins were resolved using SDS PAGE and transferred to Nitrocellulose Membrane (MDI). The membranes were blocked in 5% non fat dry milk in TBS. The membranes were washed with TBS containing 0.1% Tween 20 (TBST) and incubated in the same buffer overnight at 4°C in the presence of primary antibody (1:2000 dilution). The primary antibodies used were anti-Myc (Clontech), anti-His (Santa Cruz Biotechnology Inc), anti-HA (Sigma Aldrich), anti-GAPDH (Cell Signaling), anti-p24 (NIH) and anti-GST (Amersham Pharmacia) antibodies. The membranes were washed TBST and then incubated with secondary antibody either anti-mouse or anti-rabbit conjugated with horse radish peroxidase (1:10000 dilution, Jackson Immuno Research) in 5% non fat dry milk in TBST at room temperature. The proteins of interest were detected with EZ western horse radish peroxidase substrate (Biological Industries). GAPDH was used as loading control.

### Co-immunoprecipitation

Co-immunoprecipitation of over expressed proteins was performed using HEK 293 T cells. 6 well plates were seeded and transfected with 2 μg each of respective plasmids. 30 μM Adox (Adenosine, periodate) treatment was given to one set of cells. Cells were then harvested after 48 hours and were lysed in Lysis Buffer (20 mM Tris-HCl (pH 7.4), 40 mM NaCl, 1 mM Na_2_EDTA, 1 mM EGTA, 1% Triton X-100, 1% sodium deoxycholate, 10 mM sodium pyrophosphate, 10 mM beta-glycerophosphate, 1 mM Na_3_VO_4_, 10 mM Sodium Fluoride). The samples were sonicated on ice, centrifuged at maximum, and to the soluble fraction 30 μl of 50% Ni-NTA Beads or Glutathione Beads were added. The samples were incubated at 4°C for 2 hours while rotating and subsequently the beads were washed twice with lysis buffer and boiled in SDS-loading buffer. The proteins were separated on SDS-PAGE and immunoblotted with indicated antibodies (as described previously).

### Cycloheximide chase assay

HEK 293 T cells were transfected with plasmids encoding Myc Tat, Myc Rev, Myc ∆ Tat and Myc ∆ Rev (2 μg each). After 36 hours cycloheximide (Sigma Aldrich) was added to a final concentration of 100 μg/mL and cells were harvested after indicated time intervals. Lysate was prepared and immunoblot analysis was done as described in previously.

### Luciferase assay

HEK 293 T cells were co-transfected with pGL3 basic vector, Dicer-dependent-shLuc, pEBG, TmGST (Tat ARM cloned in pEBG), RmGST (Rev ARM cloned in pEBG). 48 hours after transfection, cell lysate was prepared using Passive Lysis Buffer (Promega) and luciferase activity was measured using a luminometer. The experiments were performed in triplicates and repeated thrice.

### Infection with HIV-1 and RNA isolation

pNL4.3 VSV-G pseudotyped virus was produced by transfecting pNL4.3 and VSV-G in HEK 293 T cells by Jet Prime Reagent (Hi Media). Virus supernatant was collected after 48 hours. Multiplicity of infection (MOI) was assessed by β-galactosidase staining of HIV-1 indicator Tzmbl cells. Jurkat T cells were infected with pNL4-3 for 4 hours at 37°C. The infected cells were harvested 48 hours after infection and subjected to immunoblotting and RNA isolation. RNA isolation was done by Trizol reagent (Sigma-Aldrich) as per manufacturer’s protocol.

### Real time PCR

Real time quantification of miRNA expression was performed using TaqMan probes specific for hiv1-miR-TAR-5p (Tar 1) and hiv1-miR-TAR-3p (Tar 2) employing the TaqMan microRNA assay kit (Applied Biosystems) according to manufacturer’s protocol. RNU6B (U6 RNA) was used as endogenous control.

## Competing interests

All authors declared that they have no competing interests.

## Authors’ contributions

SSP conceived, designed and implemented the study, working towards his PhD thesis. SA has contributed in the implementation of the work and writing of the manuscript. BK also assisted in technical work during experimentation and in manuscript preparation. ACB supervised the study and provided advice and guidance during all phases. All authors have read and approved the final manuscript.

## Supplementary Material

Additional file 1: Figure S1 DV2 NS4B Cytoplasmic Loop contains ARM like basic motif critical for RSS activity. A. shows the similarity of ARM from HIV-1 and basic motif of Dengue virus (DV) NS4B protein which makes this viral module critical to RNAi modulation. Basic LM deletion mutant in cytoplasmic loop between TMD3 and TMD4 (130- KATREAQKR -138) shows a compromised RSS function when compared with 2kNS4B and wt NS4B (B and C). Upper right panel shows FACS quantification of GFP reporter while lower half is a bar plot of three independent replicates of the above result. Dot plot depicts the number of cells (counts) on Y-axis versus the expression of GFP reporter (FL1) on X-axis. 2kNS4B and its variant NS4B are known RNAi suppressor as reported in literature (Kakumani et al., 2013). Click here for file

Additional file 2: Table S1. 10-23 DNAzyme sequences for RNAi component’s Knock down. Click here for file
