# Supplementary figures and images for: Arginine rich short linear motif of HIV-1 regulatory proteins inhibits Dicer dependent RNA interference
Source: Retrovirology. 2013 Sep 11;10:97. doi: 10.1186/1742-4690-10-97 (PMC3848888; doi:10.1186/1742-4690-10-97)

S1A

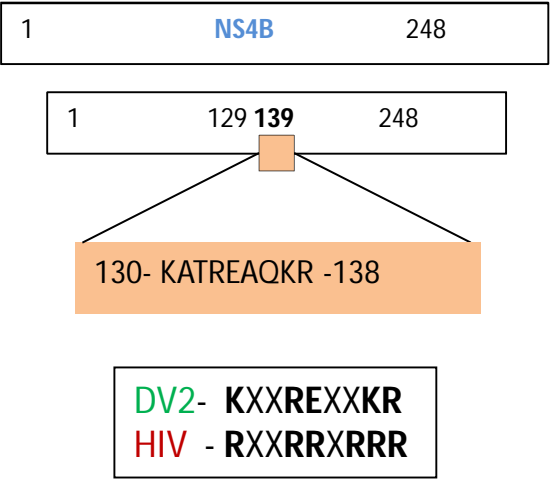

S1B

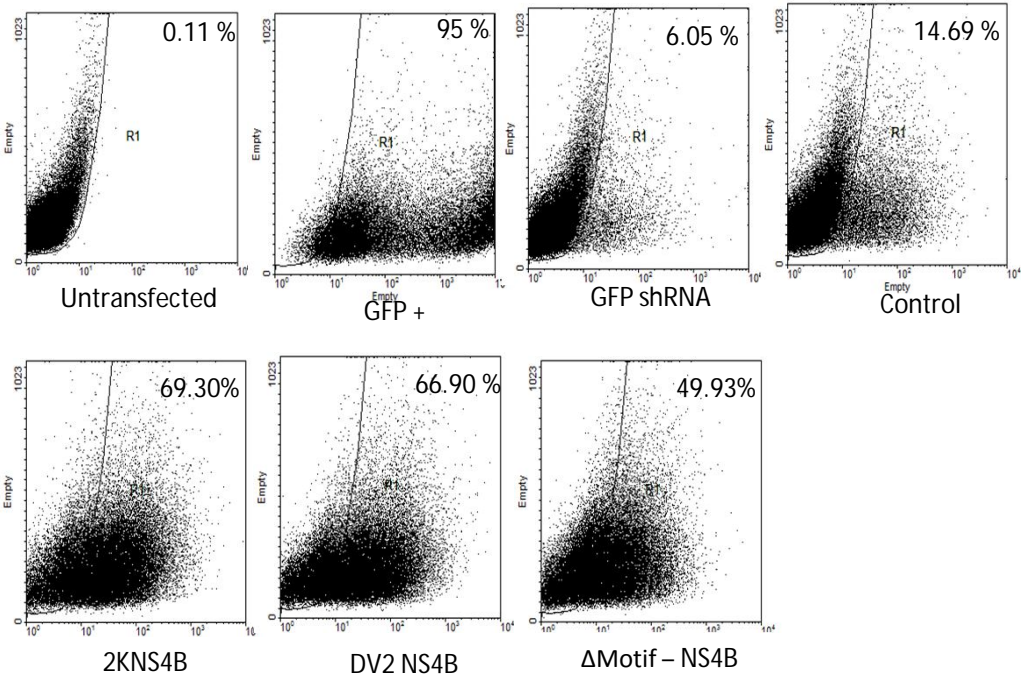

S1C

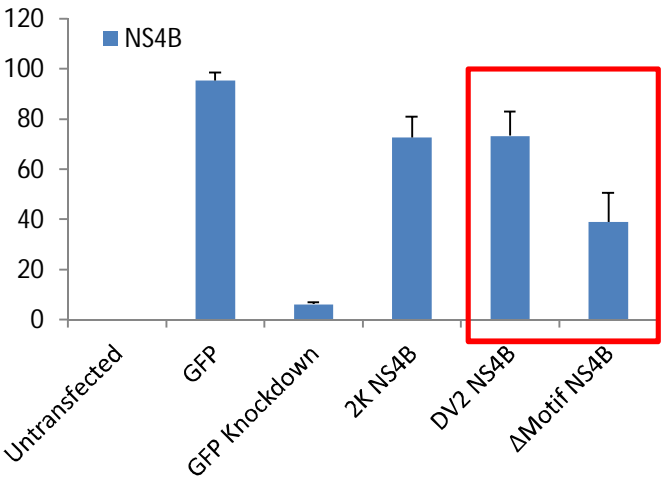

Supplement: Additional file 1: Figure S1 — DV2 NS4B Cytoplasmic Loop contains ARM like basic motif critical for RSS activity. A. shows the similarity of ARM from HIV-1 and basic motif of Dengue virus (DV) NS4B protein which makes this viral module critical to RNAi modulation. Basic LM deletion mutant in cytoplasmic loop between TMD3 and TMD4 (130- KATREAQKR -138) shows a compromised RSS function when compared with 2kNS4B and wt NS4B (B and C). Upper right panel shows FACS quantification of GFP reporter while lower half is a bar plot of three independent replicates of the above result. Dot plot depicts the number of cells (counts) on Y-axis versus the expression of GFP reporter (FL1) on X-axis. 2kNS4B and its variant NS4B are known RNAi suppressor as reported in literature (Kakumani et al., 2013). [file 1742-4690-10-97-S1.pdf]
