# Supplementary material for: Arginine rich short linear motif of HIV-1 regulatory proteins inhibits Dicer dependent RNA interference
Source: Retrovirology. 2013 Sep 11;10:97. doi: 10.1186/1742-4690-10-97 (PMC3848888; doi:10.1186/1742-4690-10-97)
Supplement: Additional file 2: Table S1. — 10-23 DNAzyme sequences for RNAi component’s Knock down. [file 1742-4690-10-97-S2.doc]

| **10-23 DNAzyme** | **Sequence 5’-3’** |
| --- | --- |
| Dzdicer1 | AGCCTTAGGCTAGCTACAACGATCTTGAA |
| Dzdicer2 | AAGCCAAGGCTAGCTACAACGATCACAGG |
| Dzdicer3 | ATTATGAGGCTAGCTACAACGACCAGAGC |
| Dzdicer4 | AGGCTGAGGCTAGCTACAACGATCTTCCC |
| DzRHA1 | GGCAGCAGGCTAGCTACAACGATGCTTTG |
| DzRHA2 | CAGGTCAGGCTAGCTACAACGATCTTAGT |
| DzRHA3 | AGTTTTAGGCTAGCTACAACGAGCTAAGA |
| DzRHA4 | TGACTGAGGCTAGCTACAACGATCCTCGA |
| DzPACT1 | AGCAGCAGGCTAGCTACAACGATCCTTTT |
| DzPACT2 | AAAGAAAGGCTAGCTACAACGAGTGGTTC |
| DzPACT3 | AAGCAAAGGCTAGCTACAACGAACTTGCA |
| DzPACT4 | GAACCAAGGCTAGCTACAACGAAGGATTA |
| DzTRBP1 | CTCAATAGGCTAGCTACAACGACCAGGTA |
| DzTRBP2 | TGCTCTAGGCTAGCTACAACGAACTAGGC |
| DzTRBP3 | TACTGAAGGCTAGCTACAACGATTCGTAG |
| DzTRBP4 | AAGACCAGGCTAGCTACAACGACCAGGCG |
| DzAgo1 | GAAGGCAGGCTAGCTACAACGAATCCTTG |
| DzAgo2 | CTTCAGAGGCTAGCTACAACGAGGACTTC |
| DzAgo3 | GATGCGAGGCTAGCTACAACGACCTTGCC |
| DzAgo4 | AGCAGGAGGCTAGCTACAACGAGTTGTTC |
